# Supplementary material for: Associations of Clusters of Cardiovascular Risk Factors with Insulin Resistance and Β-Cell Functioning in a Working-Age Diabetic-Free Population in Kazakhstan
Source: Int J Environ Res Public Health. 2023 Feb 22;20(5):3918. doi: 10.3390/ijerph20053918 (PMC10001384; doi:10.3390/ijerph20053918)
Supplement: Supplementary file 1 [file ijerph-20-03918-s001.zip › Supplementary Tables (all).pdf]

Table S1. Mean values HOMA IR and HOMA β by socio-demographic and clinical characteristics.

**Sex**	**HOMA IR**	**HOMA β**
Men	1.8651	90.2565
Women	1.9609	108.3986
p-value	0.358	0.060
**Age**		
20-29	1.8726	172.5660
30-39	1.9314	132.9460
40-49	2.0180	119.4050
50-59	2.0690	89.8365
60-69	1.6429	61.4804
p-value	0.026	0.000
**Ethnicity**		
Kazakh	1.91	101.51
Other	2.06	115.12
p-value	0.318	0.326
**Smoking**		
No	1.9210	102.6505
Yes	2.0185	106.4982
p-value	0.498	0.773
**Alcohol intake**		
No	1.9509	103.5512
Yes	1.8868	102.0231
p-value	0.543	0.876
**BMI**		
Normal	1.8788	124.1929
Overweight	1.8822	94.1755
Obesity	2.0289	93.2274
p-value	0.325	0.005
**Abdominal obesity**		
No	1.9845	124.1173
Yes	1.9047	92.6651
p-value	0.426	0.000

Table S2. Proportion of participants with Insulin Resistance and Poor β cell function by by socio-demographic and clinical characteristics.

**Sex**	**Insulin Resistance**	**Poor β cell function**
Men	21.6%	27.2
Women	23.5%	25.2
p-value	0.666	0.674
**Age**		
20-29	12.5	
30-39	23.8	13.9
40-49	23.9	13.0
50-59	27.2	24.8
60-69	15.9	59.1
p-value	0.364	0.000
**Ethnicity**		
Kazakh	23.5	26.6
Other	19.2	19.6
p-value	0.494	0.281
**Smoking**		
No	23.5	25.5
Yes	18.9	28.3
p-value	0.690	0.658
**Alcohol intake**		
No	23.5	26.8
Yes	21.7	23.3
p-value	0.448	0.465
**BMI**		
Normal	23.1	15.0
Overweight	22.1	28.3
Obesity	23.7	32.9
p-value	0.945	0.002
**Abdominal obesity**	25.2	14.0
No	21.9	31.7
p-value	0.443	0.000

Table S3. Mean values of cardiovascular factors by tertiles of HOMA IR and HOMA-β by cardiovascular risk factors.

**Cardiovascular risk factors**	**HOMA IR Tertiles**				**HOMA-β Tertiles**			
	1	2	3	p-value	1	2	3	p-value
Age	51.52	46.67	48.54	0.001	54.3	49.22	43.13	<0.000
BMI	28.17	28.26	28.482	0.893	29.92	28.51	26.48	<0.000
Waist circumference	92.89	91.1	92.8	0.463	96.57	93.22	86.97	<0.000
Hip circumference	104.56	104.28	105.38	0.711	107.62	105.51	101.09	<0.000
Systolic Blood Pressure	125.93	122.19	124.52	0.279	131.19	123.78	117.62	<0.000
Diastolic Blood Pressure	80.79	79.03	79.97	0.382	82.97	80.58	76.22	<0.000
Total cholesterol	4.8871	4.8577	4.7944	0.617	5.0206	4.9009	4.6159	<0.000
LDL Cholesterol	2.2414	2.1852	2.1403	0.495	2.3173	2.2211	2.0257	0.002
HDL Cholesterol	1.2666	1.2606	1.2806	0.874	1.22	1.3	1.29	0.082
Triglycerides	1.88	1.91	1.93	0.838	2	1.93	1.79	0.034

Table S4. Mean values of cardiovascular risk factors by Insulin resistance and Poor β cell functioning.

Cardiovascular risk factors	Insulin Resistance	p-value	Poor β-cell functioning	p-value
Age	48.28	0.534	55.51	<0.000
BMI	28.36	0.9	29.88	<0.000
Waist circumference	92.64	0.755	96.91	<0.000
Hip circumference	105.53	0.449	107.23	0.009
Systolic Blood Pressure	123.26	0.597	132.31	<0.000
Diastolic Blood Pressure	80.12	0.835	83.76	<0.000
Total cholesterol	4.77	0.284	5.07	0.001
LDL cholesterol	2.09	0.107	2.35	0.006
HDL Cholesterol	1.25	0.535	1.21	0.025
Tryglicerides	1.97	0.359	2.02	0.065
